# Emerging serotype III sequence type 17 group B streptococcus invasive infection in infants: the clinical characteristics and impacts on outcomes

**DOI:** 10.1186/s12879-019-4177-y

**Published:** 2019-06-19

**Authors:** Yi Kao, Ming-Horng Tsai, Mei-Yin Lai, Shih-Ming Chu, Hsuan-Rong Huang, Ming-Chou Chiang, Ren-Huei Fu, Jang-Jih Lu, Jen-Fu Hsu

**Affiliations:** 1 Division of Pediatric Neonatology, Department of Pediatrics, Chang Gung Memorial Hospital, Taoyuan, Taiwan; 2 Division of Neonatology and Pediatric Hematology/Oncology, Department of Pediatrics, Chang Gung Memorial Hospital, Yunlin, Taiwan; 3 Department of Laboratory Medicine, Chang Gung Memorial Hospital at Linkou, Taoyuan, Taiwan; 4 College of Medicine, Chang Gung University, Taoyuan, Taiwan; 5 Department of Medical Biotechnology and Laboratory Science, Chang Gung University, Taoyuan, Taiwan; 6 Division of Neonatology, Department of Pediatrics, Chang Gung Memorial Hospital, 5, Fu-Shing St., Kwei-Shan, Taoyuan, 333 Taiwan

**Keywords:** Group B streptococcus, Bacteremia, Drug resistance, Invasive streptococcal infection, Epidemiology

## Abstract

**Background:**

Group B Streptococcus (GBS) is an important pathogen that causes high mortality and morbidity in young infants. However, data on clinical manifestations between different GBS serotypes and correlation with molecular epidemiology are largely incomplete. The aim of this study was to determine the serotype distribution, antimicrobial resistance, clinical features and molecular characteristics of invasive GBS isolates recovered from Taiwanese infants.

**Methods:**

From 2003 to 2017, 182 non-duplicate GBS isolates that caused invasive disease in infants less than one year of age underwent serotyping, multilocus sequence typing (MLST) and antibiotic susceptibility testing. The clinical features of these infants with GBS disease were also reviewed.

**Results:**

Of the 182 patients with invasive GBS disease, 41 (22.5%) were early-onset disease, 121 (66.5%) were late-onset disease and 20 (11.0%) were late late-onset disease (> 90 days of age). All these patients were treated with effective antibiotics on time. Among them, 51 (28.0%) had meningitis, 29 (16.0%) had neurological complications, 12 (6.6%) died during hospitalization, and 15 (8.8%) out of 170 patients who survived had long-term neurological sequelae at discharge. Serotype III GBS strains accounted for 64.8%, followed by serotype Ia (18.1%) and Ib (8.2%). MLST analysis revealed 11 different sequence types among the 182 isolates and ST-17 was the most dominant sequence type (56.6%). The correlation between serotype III and ST17 was evident, as ST17 accounted for 87.3% of all serotype III isolates. There was an obvious increasing trend of type III/ST-17 GBS that caused invasive disease in infants. All isolates were susceptible to penicillin, cefotaxime, and vancomycin, while 68.1 and 65.9% were resistant to erythromycin and clindamycin, respectively.

**Conclusions:**

Despite timely and appropriate antibiotic treatment, a significant proportion of invasive GBS disease still inevitably causes adverse outcomes. Further study to explore preventive strategies and development of serotype-based vaccines will be necessary in the future.

## Background

Group B *Streptococcus* (GBS) or *Streptococcus agalactiae* is a Gram-positive coccus found in 15 to 30% of healthy women as part of normal gastrointestinal and genital tract flora [1–4]. GBS can cause invasive diseases in newborns after vertical transmission, in the elderly persons and those who have underlying immunocompromised status [5–7]. Invasive GBS infections in infants are of great concern, because a significant proportion of infants may have life-threatening meningitis, pneumonia, and bacteremia [7–11]. Recent studies also found poor long-term outcomes in GBS-infected infants, including an increased risk of death and chronic morbidities such as cerebral palsy, epilepsy, and various neurological sequelae [10–12].

Invasive GBS strains have a major virulence factor in the polysaccharide capsule, and 10 recognized capsular polysaccharides serotypes (CPS) have been identified by current molecular method, i.e., serotype Ia, Ib, and II to IX [8, 9]. Because the GBS serotype distributions and carriage rates vary greatly in different geographical areas, and the GBS carriage varies temporally [13, 14], it is important to have local and updated information regarding the molecular epidemiology of GBS strains to develop vaccines and optimize the implementation of GBS prevention algorithm [15–17]. In Taiwan, routine GBS screening has been carried out among pregnant women since 2012 and the intrapartum prophylaxis of GBS for pregnant women was also implemented in our institute since 2012. In order to obtain more precise clinical, epidemiological and molecular characteristics of invasive GBS disease, we retrospectively collected data from infants aged less than 1 year with culture-proven invasive GBS disease from the largest medical center in Taiwan. The results of serotyping, clinical characteristics, multilocus sequence typing (MLST) analysis of these GBS isolates and antimicrobial susceptibility were reported in this study.

## Methods

### GBS isolates, data collection and definition

Between January 2003 and December 2017, all young infants aged less than one year with invasive GBS diseases were enrolled and their data were retrieved retrospectively from the database of Chang Gung Memorial Hospital (CGMH). This database was filled by the clinicians. We reviewed the electronic chart records for patients’ demographics, clinical characteristics, treatment and outcomes. All GBS isolates were obtained from the bacterial library of CGMH’s central laboratory. In our institute, all positive GBS cultures were collected before antibiotics were given to infants. Early-onset disease (EOD) and late-onset disease (LOD) were defined as disease occurred between 0 and 7 days of age and between 8 and 90 days of age [6, 11, 12], respectively. Disease occurred after 90 days of age was classified as late LOD (LLOD) [6, 11, 12]. Invasive GBS disease was defined as GBS infection with GBS strains isolated from a sterile site, including blood, urine, cerebrospinal fluid (CSF), soft tissues (necrotic tissues, abscesses, or cellulitis), pleural or peritoneal fluids. We did not enroll GBS isolates from sputum or bronchoalveolar lavage fluid. This study was approved by the ethics committee of CGMH (IRB No. 104-6818B), and a waiver of informed consent for anonymous data collection was approved.

Meningitis was defined by the World Health Organization as the presence of clinical signs of possible serious bacterial infection [18] and CSF culture positive for bacterial pathogens or blood culture/polymerase chain reaction (PCR)/latex agglutination positive for bacterial pathogens with a CSF leukocyte count > 20 × 10^6^/L. Episodes reported by physicians with negative CSF cultures were also included if CSF results showed at least one individual marker of bacterial meningitis (defined as a glucose level of less than 34 mg/dL [1.9 mlol/L], a ratio of CSF glucose to blood glucose of less than 0.23, a protein level of more than 220 mg/dL, or a leukocyte count of more than 2000/μL) [19] and the clinical presentation was compatible with bacterial meningitis. In this study, the definitions of bacteremia, pneumonia and septic shock were also based on the Center of Disease Control [20]. We also evaluated the presences of neurological complications and long-term neurological sequelae of these patients, based on the definition of previous studies [12, 21].

### Capsular serotyping

The capsule genotypes of all isolates were analyzed using the multiplex PCR approach, and this assay, as well as the DNA isolation method, was described in our previous publication [22].

### MLST assay and assignment to clonal clusters

MLST was carried out as described in our previous study [23]. Briefly, PCR was used to amplify fragments from seven housekeeping genes (*adhP*, *atr*, *glcK*, *glnA*, *pheS*, *sdhA*, and *tkt*), and then seven PCR products were purified and sequenced. The sequence type (ST) was assigned based on the allelic profile of each fragment and determined via the *Streptococcus agalactiae* MLST database (http://pubmist.org/sagalactiae).

### Antimicrobial susceptibility testing

Antimicrobial susceptibility testing was performed with the disc diffusion method as described in previous studies [24]. The double-disk diffusion test was applied to identify inducible clindamycin resistance. All GBS isolates were rated for susceptibility to seven antibiotics, including erythromycin, penicillin, clindamycin, vancomycin, ampicillin, cefotaxime and teicoplanin according to the guidelines of the Clinical and Laboratory Standards Institute for the microdilution minimum inhibitory concentration (MIC) method [25].

### Statistical analysis

CPS distribution with respect to EOD or LOD, gender, and clinical characteristics including outcomes were compared using the χ^2^ test. To assess the differences between nominal variables, the Student’s t test or the Mann-Whitney *U* test was used. The trend of proportions of the categorical variables among the three groups was analyzed by the Cochran-Armitage trend test. *P* values of < 0.05 were considered statistically significant. Data were analyzed using SPSS version 23 (IBM SPSS Statistics).

## Results

### CPS distribution and clinical characteristics

From January 2003 to December 2017, 182 nonduplicate GBS isolates from neonates with invasive GBS (iGBS) disease in CGMH were collected, and clinical characteristics were recorded. There were a total of 41 EOD (22.5%), 121 LOD (66.5%), and 20 LLOD (11.0%) (Table 1). Serotypes III predominated in these invasive GBS strains (*n* = 118, 64.8%), followed by serotype Ia (*n* = 33, 18.1%) and Ib (*n* = 15, 8.2%). Only 6 serotypes were detected during the 15-year study period surveyed. The majority of iGBS isolates (85.7%) were submitted from blood specimens, and only a few isolates were collected from CSF (24 cases [13.2%]). Both pleural fluid and intra-abdominal source each accounted for one case (0.55%), respectively.

**Table 1:** Numbers of invasive group B Streptococcal (iGBS) infections cases by capsular polysaccharide serotypes (CPS) and the clinical characteristics

CPS	Age of iGBS onset			Source of iGBS isolates			
	EOD	LOD	LLOD	Blood	CSF	Ascites	Pleural fluid
Ia	10 (24.4)	19 (15.7)	4 (20.0)	27 (17.3)	6 (25.0)	0 (0)	0 (0)
Ib	10 (24.4)	2 (1.7)	3 (15.0)	13 (8.3)	2 (8.3)	0 (0)	0 (0)
II	2 (4.9)	1 (0.8)	0 (0)	3 (1.9)	0 (0)	0 (0)	0 (0)
III	14 (34.1)	96 (79.3)	8 (40.0)	102 (65.4)	16 (66.7)	0 (0)	0 (0)
V	2 (4.9)	2 (1.7)	2 (10.0)	6 (3.8)	0 (0)	0 (0)	0 (0)
VI	3 (7.3)	1 (0.8)	3 (15.0)	5 (3.2)	0 (0)	1 (100)	1 (100)
Total	41 (22.5)	121 (66.5)	20 (11.0)	156 (85.7)	24 (13.2)	1 (0.55)	1 (0.55)

EOD: early onset disease (≤ 7 days); LOD: late onset disease (8–90 days); LLOD: late late-onset disease (> 90 days); CSF: cerebrospinal fluid

**Fig. 1:**
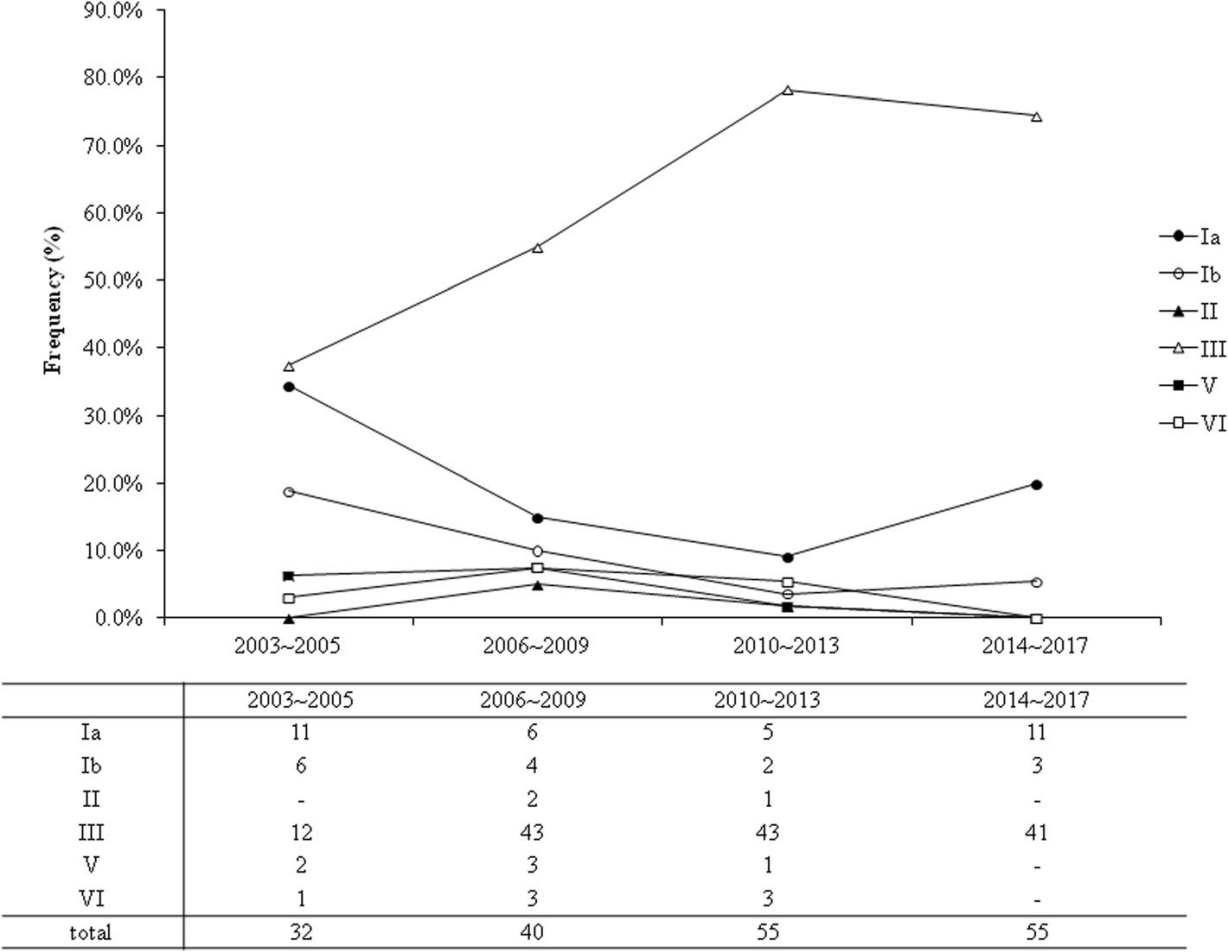
The percentage of six serotypes invasive group B streptococcal disease in infants younger than one year old in Taiwan during a 15-year study period

When comparing the distribution of serotypes in LOD and EOD, type III was significantly predominant in LOD (96 cases, 79.3%, *p* < 0.001 by χ^2^ test) and type Ib was significantly common in EOD (10 cases, 66.7%). Other serotypes were similarly distributed. In this 15-year study period, we found a significant increase in serotype III in young infants with invasive GBS diseases (Fig. 1). Serotype III accounted for 37.5% of all invasive GBS strains during 2003–2005, and increased to 78.2 and 74.5% of all invasive GBS isolates during 2010–2013 and 2014–2017, respectively (both *p* < 0.001).

The clinical presentations of these invasive GBS diseases are summarized in Table 2. The majority of these neonates were term-born (gestational age [GA] more than 37 weeks, 79.1% [144/182]), and only 8 (4.4%) were GA < 30 weeks. The median birth weight was 2900 g (interquartile range: 2557-3300 g), and 57.7% were male. Of the total 182 cases, 12 (3 EOD, 7 LOD, and 2 LLOD) died before discharge, with a mortality rate of 6.6%, and 29 (15.9%) had neurological complications. Among those who survived and with neurological complications, 14 (45.4%) had long-term neurological sequelae at discharge. Although serotype III GBS strains accounted for more than half of neonatal meningitis (31/51, 60.8%), infants with type III GBS invasive disease were significantly less severe and had relatively better prognosis, including lower risk of neurological complications, less sepsis-attributable mortality and a higher rate of complete cure without any sequelae at discharge. On the contrary, infants with type Ib and type V GBS diseases had a significantly higher rates of respiratory failure, disseminated intravascular coagulopathy (DIC), and multi-organ failure (all *P* values were < 0.05 by *x*^2^ test with Bonferroni correction) when compared with other serotypes. Furthermore, infants with type Ib and V GBS invasive disease had the highest risk of mortality (both 33.3%, *P* < 0.001).

**Table 2:** Demographics and clinical features of neonates with serotype III GBS invasive infections compared with those with other serotype GBS invasive infections

Clinical characteristics	Type III iGBS (*n* = 118)	Type Ia iGBS (*n* = 33)	Type Ib iGBS (*n* = 15)	Type V iGBS (*n* = 6)	Other iGBS (*n* = 10)
Gestational age (weeks), median (IQR)	39.0 (38.0–40.0)	38.0 (37.0–39.0)	37.0 (30.0–39.0)*	35.5 (29.8–39.3)*	37.0 (34.8–40.0)
Birth body weight (g), median (IQR)	3040 (2700–3350.0)	2760 (2490–3085)	3015 (1350–3165)*	2248 (1586–2912)*	2560 (2016–3138)*
Gender, male/female n (%)	77 (65.3)/41 (34.8)	13 (39.4)/20 (60.6)	8 (53.3)/7 (46.7)	4 (66.7)/2 (33.3)	5 (50.0)/ 5 (50.0)
Onset of diseases (day), median (IQR)	27.5 (14.8–53.3)	30.0 (3.5–56.5)	2.0 (1.0–30.0)*	14.0 (1.0–21.3)*	2.5 (1.0–11.8)*
Early-onset disease (≤ 7 days)	14 (11.9)	10 (30.3)	10 (66.7)*	2 (33.3)	5 (50.0)
Late-onset disease (8–90 days)	96 (81.4)*	19 (57.6)	2 (13.3)	2 (33.3)	2 (20.0)
Late late-onset disease (> 90 days)	8 (6.8)	4 (12.1)	3 (20.0)	2 (33.3)	3 (30.0)
Clinical manifestations					
Bacteremia	115 (97.5)	32 (97.0)	15 (100)	6 (100)	10 (100)
Meningitis	31 (26.3)	13 (39.4)	6 (40.0)	0 (0)	1 (10.0)
Pneumonia	12 (10.2)	2 (6.1)	5 (33.3)	0 (0)	2 (20.0)
Septic shock	8 (6.8)	9 (27.3)	7 (46.7)**	3 (50.0)**	4 (40.0)*
Respiratory failure	8 (6.8)	8 (24.2)	9 (60.0)**	4 (67.7)**	4 (40.0)*
Multi-organ failure	2 (1.7)	3 (9.1)	7 (46.7)**	3 (50.0)**	4 (40.0)*
Disseminated intravascular coagulopathy	5 (4.2)	4 (12.1)	7 (46.7)**	3 (50.0)**	4 (40.0)*
Treatment outcomes					
Complete cure without any sequelae at discharge	104 (88.1)	26 (78.8)	8 (53.3)**	3 (50.0)*	7 (70.0)
Neurological complications	15 (12.7)	6 (18.2)	2 (13.3)	1 (16.7)	5 (50.0)**
Mortality	1 (0.8)	3 (9.1)	5 (33.3)**	2 (33.3)**	1 (10.0)

All data were expressed as number (percentage %), unless indicated otherwise

IQR: interquartile range

*P < 0.05 by *x*^2^ test with Bonferroni correction

**P < 0.001 by *x*^2^ test with Bonferroni correction

**Fig. 2:**
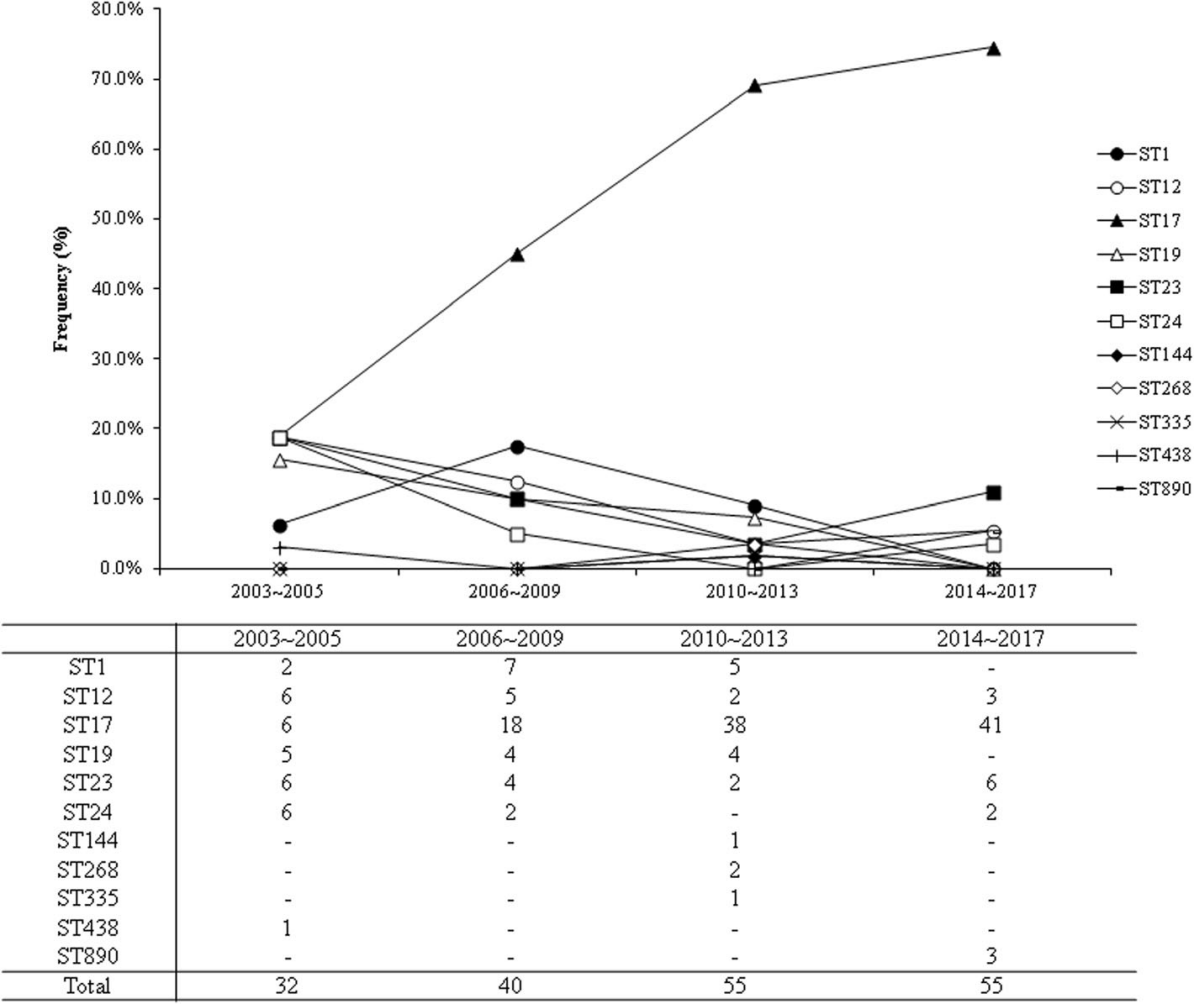
Invasive group B streptococcal disease in young infants younger than one year old in Taiwan, 2003–2017, stratified based on different sequence types and time period

All these isolates underwent MLST analyses. The MLST analyses revealed 11 different STs among the 182 GBS isolates. ST17 was the predominant ST and accounted for 56.6% of all isolates. All STs have been identified in the GBS MLST database (http://pubmist.org/sagalactiae). Relationships between STs and GBS serotypes of the 182 isolates characterized in this study are presented in Table 3. The correlation between serotype III and ST17 was evident, as ST17 accounted for 87.3% (*n* = 103, 95% confidence interval [CI] 86.6–88.0%) of all serotype III isolates. The other 15 serotype III isolates belonged to ST19 (11.0%, *n* = 13), ST335 (0.8%, n = 1), and ST438 (0.8%, n = 1). Furthermore, the increasing trend of ST17 was also obvious, as we found that ST17 accounted for only 18.8% of all invasive GBS diseases during 2003–2005, and this strain increased to 69.1 and 74.5% of all GBS isolates during 2010–2013 and 2014–2017, respectively (both *p* < 0.001) (Fig. 2).

**Table 3:** Relationships between sequence type and serotype in 182 invasive GBS isolates in CGMH, 2003–2017

Sequence type	Serotype						
	Ia (%)	Ib (%)	II (%)	III (%)	V (%)	VI (%)	Total (%)
ST1	1	–	3	–	3	7	14 (7.7)
ST12	–	15	–	–	1	–	16 (8.8)
ST17	–	–	–	103	–	–	103 (56.6)
ST19	–	–	–	13	–	–	13 (7.1)
ST23	16	–	–	–	2	–	18 (9.9)
ST24	10	–	–	–	–	–	10 (5.5)
ST144	1	–	–	–	–	–	1 (0.5)
ST268	2	–	–	–	–	–	2 (1.1)
ST335	–	–	–	1	–	–	1 (0.5)
ST438	–	–	–	1	–	–	1 (0.5)
ST890	3	–	–	–	–	–	3 (1.6)
Total (%)	33	15	3	118	6	7	182 (100)

*ST* sequence type

All isolates were susceptible to penicillin, ampicillin, and vancomycin. None of these strains displayed cefotaxime resistance. The overall rates of erythromycin and clindamycin resistance were 68.1 and 65.9%, respectively. Most of the GBS isolates that were erythromycin resistant (*n* = 124, 68.1%) were also clindamycin resistant (94.4%, 117 isolates, *p* < 0.001 by Pearson χ^2^ test). As we observed antimicrobial resistance profiles by serotype and ST, significantly higher rates of erythromycin and clindamycin resistances were noted in serotypes Ib (100%), III (77.1–82.2%) and V (66.7%). There was no significant difference in the numbers of erythromycin-nonsusceptible or clindamycin-nonsusceptible GBS isolates obtained from groups of EOD, LOD, or LLOD. Because the high correlation between ST17 and type III, and ST 12 and type Ib GBS strains, erythromycin resistance was found to be especially high among ST12 (100%) and ST17 (89.3%).

## Discussion

Current studies of GBS have focused on the source, the bacterial factors influencing the transmission of GBS from mother to infants, and the predisposing factors for invasive GBS late-onset sepsis (LOS) in colonized infants [26–28]. Other studies have emphasized the need to develop GBS vaccines [29, 30]. Therefore, all these studies highlighted the importance of preventive strategies and were based on epidemiological and molecular characteristics of GBS [24–31]. In this study, we also characterized the clinical manifestations of various invasive GBS strains in infants. We found a predominant and increasing trend of type III/ST-17 GBS strains in the past 15 years. In our cohort, although type III GBS strains accounted for more than half of neonatal meningitis, it appeared to be unrelated to the worst prognosis. On the contrary, type Ib and type V GBS diseases were associated with the highest illness severity and had the worst treatment outcomes. Type Ib and type III GBS strains had significantly higher resistance to erythromycin and clindamycin.

Serotype III GBS has become the most prevalent strain in Asia, accounting for 32.2–77.9% of all invasive GBS isolates in various countries and populations of specific age [32–36]. Although the distribution and predominance of certain serotypes are susceptible to variations and may change over time, there is an increasing trend of serotype III in infants [37, 38], which is also observed in our cohort. Furthermore, serotype III GBS is the most dominant invasive clone accounting for the majority of LOD cases with meningitis [35, 38, 39]. The most prevalent ST found in our cohort was ST17, followed by ST23 and ST12. The common ST19 strain found in other countries only accounted for 7.1% of our isolates. In colonized pregnant women and adults with invasive disease, most type III GBS isolates belong to ST-17 and ST-19 [35, 39, 40]; however, serotype III GBS strains that cause invasive disease in infants are almost exclusively ST-17 lineage. These results indicate that neonatal invasive diseases may not be directly derived from maternal colonization, and the transmission routines of GBS LOD, especially those occur later than 90 days of age (late LOD), remain unknown.

In this present study, it is noteworthy that type Ib, type V and type VI GBS strains were associated with more severe clinical presentations, including higher rates of septic shock, disseminated intravascular coagulopathy, and multi-organ failure, leading to a higher mortality. Besides, these strains were associated with a higher rate of neurological complications, although only type Ib GBS strain often caused meningitis. These complications resulted from poor cerebral perfusion during septic shock rather than sequelae related to meningitis. The higher mortality rate of type V GBS resulted from the lower gestational age (3 out of 6 were preterm infants, including 2 less than 28 weeks). On the contrary, although type III GBS strains were associated with a higher rate of meningitis, most were completely cured at discharge. Few studies have characterized the clinical manifestations of invasive GBS diseases in a specific serotype or sequence type, but our findings were limited by small sample sizes. Therefore, further systemic reviews or multi-center studies are required to conclude these clinical features.

The overall mortality in our cohort was 6.6%, which was lower than the average mortality of 8.4% (95% confidence interval 6.6–10.2) reported by other countries [11], including Japan, Korea, India, and a recent study in South China [30, 36, 41–43]. In our cohort, 28% (total *n* = 51) had CSF culture proven or clinical meningitis, which was significantly higher than previous reports [11, 36, 41–43]. Furthermore, the presence of neurological sequelae after treatment in our cohort (16%) was significantly lower than the reported 25–35% in previous studies [43, 44]. These may result from different definitions of meningitis and neurological sequelae. Because none of these isolates were resistant to penicillin and none had delayed administration of effective antibiotic when disease onset, further study to explore optimized therapeutic strategies will be necessary given the remarkable adverse outcomes in these patients.

The rates of GBS resistance to macrolide and clindamycin have been increasing in a global trend since the 2000s [45, 46]. It is noteworthy that GBS isolates resistant to vancomycin and gentamicin have recently been reported in some geographies [47, 48]. In this cohort, certain GBS serotypes and STs were significantly associated with erythromycin and clindamycin resistance. The increasing trend of GBS resistance to macrolide and clindamycin in our cohort is associated with the trend of type III/ST-17 GBS isolates. Furthermore, type Ib GBS has the highest rate of antimicrobial resistance, which is in consistent with that reported in Southeast Asia [47–49]. GBS isolates in Taiwan and China have been found to have a higher rate of macrolide resistance (58–70.8%), when compared with the resistant rate of 11.5–36% reported in Europe and North America [11, 37, 40, 41, 49–52]. These findings add concerns regarding the prophylactic use of ampicillin in pregnant women, as antibiotic selection may account for the increased antibiotic resistance, which finally lead to limited therapeutic choices for penicillin-intolerant patients.

This study has some limitations. All GBS isolates were from a single center in Taiwan, and may not represent the whole epidemiological features of this geographic area. Most of our iGBS strains were type III/ST-17, and other serotypes, especially type II, V and VI were small case numbers. Therefore, we were unable to characterize the clinical manifestations of specific serotype GBS invasive diseases. The slide agglutination was not performed in this study, so the expression of genes detected was unknown. Furthermore, although we found that the increasing trend of type III/ST-17 GBS strains is causing invasive diseases in infants, we did not have data of population-based incidence rate. Finally, some false-negative cases or early mortality caused by GBS infection might have been missed and were not included in this study.

## Conclusions

In conclusion, this study demonstrates that GBS remains one of the most important pathogens that cause neonatal mortality and morbidity, especially those with meningitis and neurological sequelae. There is an increasing trend of type III/ST-17 GBS in Taiwan, and the increasing trends of antibiotic resistance to erythromycin and clindamycin were also noted in Taiwan, and in Asia. This is of great concern, because the antibiotic options for penicillin-allergic women will be limited. Furthermore, we used to have initial appropriate antibiotic treatment in time, but the adverse outcomes of invasive GBS diseases are sometimes inevitable. Therefore, further study on more aggressive treatments and monitoring will be necessary in the future.

## Data Availability

The datasets used/or analyzed during the current study available from the corresponding author on reasonable request.

## Abbreviations

CGMH: Chang Gung Memorial Hospital
CPS: Capsular polysaccharides serotypes
CSF: Cerebrospinal fluid
EOD: Early-onset disease
GBS: Group B streptococcus
LLOD: Late late-onset disease
LOD: Late-onset disease
MLST: Multilocus sequence typing
ST: Sequence type
